# Comparative effectiveness of ultrasound and paraffin therapy in patients with carpal tunnel syndrome: a randomized trial

**DOI:** 10.1186/1471-2474-15-399

**Published:** 2014-11-26

**Authors:** Yi-Wei Chang, Shih-Fu Hsieh, Yu-Shiow Horng, Hui-Ling Chen, Kun-Chang Lee, Yi-Shiung Horng

**Affiliations:** 1 Department of Physical Medicine and Rehabilitation, Taipei Tzuchi Hospital, Buddhist Tzuchi Medical Foundation, New Taipei City, Taiwan; 2 Department of Medicine, Tzu Chi University, Hualien, Taiwan; 3 Department of Physical Medicine and Rehabilitation, Keelung Hospital Ministry of Health and Welfare, Keelung City, Taiwan

**Keywords:** Carpal tunnel syndrome, Paraffin therapy, Ultrasound therapy

## Abstract

**Background:**

Conclusive evidence indicating an effective treatment for carpal tunnel syndrome (CTS), a common entrapment neuropathy, is lacking. Ultrasound therapy (US therapy) has long been used as one of the combination treatments for CTS. In addition, paraffin bath therapy has been applied widely as a physical modality in treating patients with hand conditions. The purpose of this randomized trial was to compare the efficacy of combining a wrist orthosis with either US therapy or paraffin bath therapy in treating CTS patients.

**Methods:**

Patients with CTS were randomized into two groups. All patients received a wrist orthosis. Twice per week, one group underwent paraffin therapy, and the other group underwent ultrasound therapy. Each patient received a questionnaire, physical examination and nerve conduction study of the upper extremities before and after treatment for eight weeks.

**Results:**

Sixty patients were recruited, and 47 completed the study. Statistical analysis revealed significant improvements in symptom severity scores in both groups. After adjusting for age, gender and baseline data, the analysis of covariance revealed a significant difference in the functional status score between two groups.

**Conclusions:**

The combination of ultrasound therapy with a wrist orthosis may be more effective than paraffin therapy with a wrist orthosis.

**Trial registration:**

Clinicaltrial.gov: NCT02278289 Oct 28, 2014

**Electronic supplementary material:**

The online version of this article (doi:10.1186/1471-2474-15-399) contains supplementary material, which is available to authorized users.

## Background

Carpal tunnel syndrome (CTS) is a common entrapment neuropathy that causes symptoms of pain, numbness and paresthesia in the distribution of the median nerve and may even cause atrophy of the thenar muscle [1, 2]. For patients with mild to moderate symptoms, nonsurgical treatments, such as local steroid injection, oral medication, wrist orthoses, therapeutic exercise, ultrasound therapy (US therapy), low-level laser and paraffin bath have been implemented clinically [1, 3, 4]. However, conclusive evidence on the best treatment for patients with CTS is lacking.

For years, US therapy has been used as one of the combination treatments for CTS [1–3, 5]. The mechanism of US therapy includes thermal and nonthermal effects. The thermal effect occurs when acoustic waves penetrate the tissue and produce molecular vibration, which results in heat production and facilitates pain relief. [6] The nonthermal effect of US therapy includes cavitation, media motion and standing waves, which might elicit anti-inflammatory and tissue-stimulating effects [7, 8]. Several clinical trials have revealed US therapy has a positive effect on patients with CTS [5, 9]. However, Cochrane’s 2013 review concluded that there is still insufficient evidence to support that US therapy is more effective than placebo or other nonsurgical interventions for CTS [10]. Additional research is still needed to compare the effectiveness of US therapy with other modalities for patients with CTS, particularly in the long term.

Paraffin therapy has been widely used as a physical modality in treating patients with hand conditions, such as rheumatoid arthritis, osteoarthritis and CTS [4, 11, 12]. Paraffin therapy provides superficial heat to the hands, which can both relieve pain and improve local circulation [6, 13]. Previous studies have revealed that paraffin therapy could improve pain and finger joint range of motion in patients with hand arthritis [11, 12]. Symptom improvements were also observed in patients with CTS after receiving combination treatments with paraffin therapy and a wrist orthosis [4]. However, to the best of our knowledge, no previous clinical trial has compared the effectiveness of paraffin bath with US therapy for CTS patients.

### Purpose

The purpose of this exploratory study is to compare the combination of a wrist orthosis with either US therapy or paraffin bath therapy in the treatment of CTS patients. We hypothesized that US therapy might be more effective than paraffin therapy because it provides both thermal and nonthermal effects.

## Methods

### Patients and controls

The Institutional Review Board of our hospital (Taipei Tzuchi Hospital Institutional Review Board Committee) approved this study, and patients provided informed consent prior to the study. Sixty individuals diagnosed with CTS were recruited from the Department of Physical Medicine and Rehabilitation in one community hospital during 2010 and 2011. Study inclusion criteria required patients to have subjective symptoms (such as pain and/or numbness in the median nerve distribution of the digits or nocturnal pain). Furthermore, patients were required to have either a positive Phalen’s sign or a positive Tinel’s sign along with electrophysiological evidence of CTS. We excluded patients with (1) age younger than 18 years old; (2) underlying medical disorders, such as diabetes mellitus, renal failure, autoimmune disease or hypothyroidism; and (3) pregnancy, previous wrist trauma or surgery.

**Figure 1:**
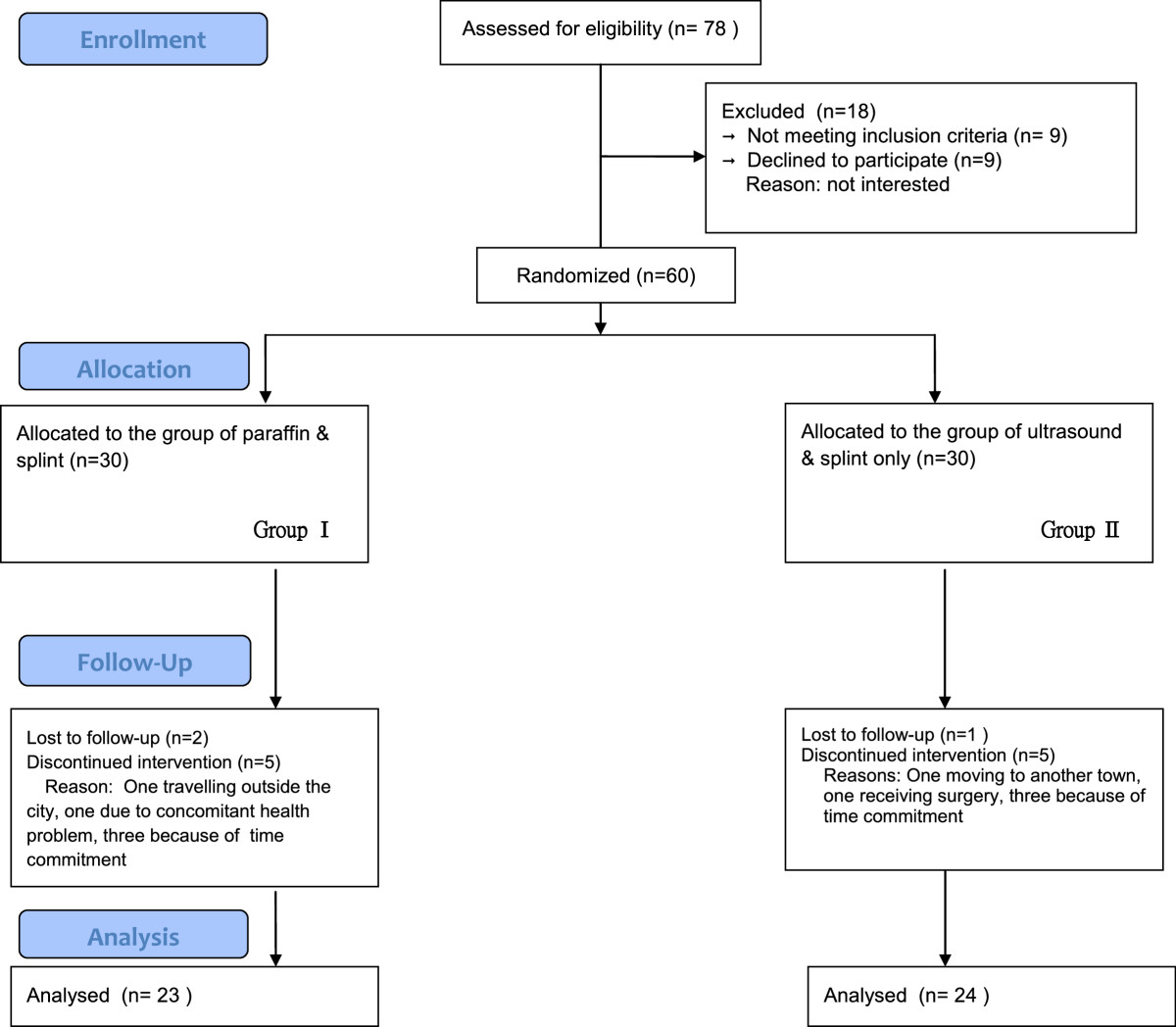
**Flowchart of patients’ recruitment.**

All eligible patients were invited, and the participants were randomly assigned to two groups. A total of 60 lots were prepared with 30 lots for each group, and each lot was sealed in a non-transparent envelope with the same appearance. All envelopes were randomly mixed together numerous times. Finally, the envelopes were marked from 1 to 60 by an assistant who was not involved in the mixing process, and the study nurse simply picked up the lot sequentially. The allocations were concealed with the use of packages of prescription orders, which were given by the nurse to the physical therapists, and the therapy programs were administered by physical therapists who did not participate in evaluating the study outcome.The participants were randomly allocated into two groups. One group received paraffin therapy and a wrist orthosis, and the other group received US therapy and a wrist orthosis. Custom-made neutral wrist orthoses were given to all the patients, who were instructed to wear the wrist orthoses while sleeping for at least eight weeks. A CONSORT flowchart describing the process of participant randomization and intervention is depicted in Figure 1.

A series of physical examinations and nerve conduction studies (NCSs) were performed on each patient. Physical examination included the palmar pinch power test, the Semmes-Weinstein Monofilament sensory test, Tinel’s test and Phalen’s test. Participants completed a set of questionnaires, including the Boston CTS questionnaire and several questions involving basic demographic information. Numbness and pain were assessed using a 10-cm visual analog scale (VAS).

After receiving the designated 8-week therapy, all patients were re-evaluated using the same clinical examinations, questionnaires and NCSs. The outcomes of the physical examination and the NCSs were assessed by physiatrists who were not aware of the group assignments.

### Paraffin therapy

Patients in the paraffin therapy group were treated with the dip-and-wrap method of paraffin bath therapy in the hospital twice per week for 8 weeks. The temperature of the paraffin bath was maintained at approximately 55°C (Parabath, Hygenic Corporation, Akron, OH, USA). The whole procedure is described as follows. Patients dipped their affected hands into the paraffin wax. Next, they waited for the paraffin wax to harden and then dipped their hands again into the paraffin wax. This step was repeated 5 times. When the last paraffin layer hardened, it was covered with plastic wrap and a towel. After 20 minutes of heating, the paraffin was removed [12].

### Ultrasound therapy

Patients in the ultrasound group were treated with US therapy for 5 minutes each session, twice per week for 8 weeks. The US machine was set at a frequency of 1 MHz, an intensity of 1.0 W/cm^2^, in pulsed mode (1:4) with a transducer 5 cm^2^ in size (Therasound 3.5, Rich-Mar Corporation, Inola, OK, USA), and with aquasonic gel as couplant [3]. The transducer was placed over the wrist carpal tunnel area, ranging from wrist crease to palmar region. A stroking method was used with a sonation area of approximately 5 × 5 cm^2^. The machine was calibrated, and the output was adjusted regularly with a simple underwater balance.

### Outcome measurements

The patients were evaluated with the Boston CTS questionnaire, a pain scale, physical examinations and NCSs before and after treatment for eight weeks.

### Primary outcome

The primary outcome was the functional status scale of the Boston CTS questionnaire. The Boston CTS questionnaire is a self-administered outcome measurement for CTS patients, consisting of two parts: a symptom severity scale (11 questions) and a functional status scale (8 questions). All of the answers were scored from 1 to 5 according to the patient’s clinical condition, such that 1 indicated no symptoms, and 5 indicated the most severe symptoms. The questionnaire’s reproducibility, internal consistency and responsiveness were validated in the previous paper [14]. The functional status scale of the Boston CTS questionnaire was chosen as the primary outcome because it is closely correlated with the patient’s ability to perform daily activities. The goal of rehabilitation is to improve the functional status of patients, rather than only symptom relief.

### Secondary outcomes

The secondary outcomes were the symptom severity scale of the Boston CTS questionnaire, the pain scale, changes in the monofilament sensory test, palmar pinch power and the distal sensory and motor latencies of the median nerve.

### Physical examinations

Phalen’s test was performed by asking the patients to fully flex their wrist for 60 seconds. A positive test occurred when patients experienced symptoms of numbness and tingling in the median nerve distribution [15]. Tinel’s sign was elicited by gently tapping the median nerve at wrist level. This test was considered positive when patients reported signs of a tingling sensation or shooting pain along the median nerve distribution of the hand [16]. Palmar pinch strength was measured by pressing the thumb and the index finger tip against a standard dynamometer. This procedure was repeated 3 times, and a mean score was obtained [17].

The Semmes-Weinstein monofilament sensory test was measured by applying force-calibrated nylon filament to the fingertips with the wrist in a neutral supine position. Each type of filament was pressed perpendicularly against the fingertips until the filament bent into a C shape. This examination was considered positive if the patient was able to correctly identify which digit the monofilament was touching with his/her eyes closed. A weighted score from 1 to 5 was acquired according to each filament’s calculated force [14]. We recorded the scores from seven sample areas in each hand and summed the scores to analyze as a continuous variable.

### Nerve conduction study

Median and ulnar nerve sensorimotor NCSs were conducted on all patients utilizing Neuropack M1 MEB-9200 J/K electrodiagnostic equipment (Nihon Kohden Corporation, Tokyo, Japan) in a quiet, air-conditioned room (26°C). The patients were prepared in the supine position with their skin temperature measured on the palms and maintained above 32°C. Standard techniques of supramaximal percutaneous stimulation with a constant current stimulator and surface recording were used for the NCS. Median motor nerve conduction and distal motor latency were measured by placing a stimulating electrode at the wrist and a recording electrode on the abductor pollicis brevis muscle 8 cm from the stimulus electrode. A standard distance (14 cm) was maintained between the stimulator and recording electrodes for the sensory nerve conduction studies [18]. The ring finger difference was calculated as the median nerve peak latency minus the ulnar nerve peak latency [19]. The diagnosis of CTS was established if at least one of three criteria was achieved: (1) distal motor latency >4.4 milliseconds, (2) distal sensory latency >3.4 milliseconds [20] or (3) median-ulnar distal sensory latency difference (ring difference) >0.4 milliseconds [19].

### Sample size

For sample size estimation, previous randomized, controlled trial studies, conducted in CTS patients receiving carpal tunnel injection, suggested that 26 subjects per group would provide 90% statistical power and a 5% significance level by two-sided tests to detect a significant decrease in the Boston CTS questionnaire score from 1.6 to 2.0 [21, 22]. To compensate for a 15 to 20% dropout rates, we recruited 30 subjects per group.

### Statistical analysis

The following data were analyzed: (1) descriptive statistics to summarize the participants' basic demographics; (2) the baseline and follow-up scores for patient-reported outcomes (PROs; including the symptom severity scale, the functional status scale and pain intensity), using paired t-tests for each patient; (3) the differences in changes in the PROs after treatment between the groups by analysis of covariance (ANCOVA), with adjustments for age, sex and the baseline data for each item before treatment to accommodate individual differences; (4) the baseline and follow-up physical examinations and NCS data for each affected hand, using the generalized estimate equation (GEE) method, which is a quasi-likelihood approach for correlated data that does not fully specify the distribution of responses in each cluster, while considering that these examinations were performed on both hands for those patients who had bilateral CTS; this GEE method was applied with the subjects as clusters, and in this model, the two hands of each individual were treated as correlated [23]; and (5) the differences in changes in the physical examination and NCS data between the two studied groups using the GEE model, with age, sex and baseline values as covariates. In addition, we calculated the effect size (ES; mean changes in scores divided by baseline standard deviation) for PROs. All of the statistical analyses were performed using the SAS statistical software package, version 9.2. (SAS Institute Inc., Cary, NC, USA).

## Results

### Patient characteristics and patient-reported outcomes

Seventy-eight patients were enrolled in this study, and 18 patients were excluded after being assessed for eligibility. Among the excluded patients, nine did not meet the inclusion criteria and nine declined to participate. A total of 60 patients with CTS were recruited and randomized into the two study groups. Forty-seven patients completed the study. Seven and six patients were unable to complete the study in the paraffin and US therapy groups, respectively (Figure 1). Table 1 summarizes the demographic characteristics and basic participant information. As shown in Table 1, the mean ages of the patients in the paraffin and US therapy groups were 51.9 ± 8.8 and 48.8 ± 11.2 years, respectively. More than half of the patients were female and had bilateral CTS.

**Table 1:** **Frequency distributions (percentages) of demographic characteristics in patients with CTS who completed the study**

Characteristics	Treatment group	
	Paraffin therapy	US therapy
	n = 23, n (%)	n = 24, n (%)
**Personal characteristics**		
Age, mean±SD, yrs	51.9±8.8	48.8±11.2
Body mass index, mean±SD	25.7±4.5	25.0±3.7
Male	3 (13.0)	2 (8.3)
Married	17 (77.3)	17 (70.8)
Employed	10 (40.5)	14 (58.3)
Smoking habit	0 (0.0)	1 (4.2)
Right-hand dominant	21 (95.5)	23 (100.0)
Bilateral hands involved	20 (87.0)	17 (70.8)
**Educational level**		
College/University	7 (30.4)	9 (37.5)
Senior High	8 (34.8)	11 (45.8)
Junior High or below	8 (34.8)	4 (16.7)
**Household monthly income (US$)**		
<1200	8 (34.8)	3 (12.5)
1200-3500	10 (43.5)	16 (66.7)
>3500	5 (21.7)	5 (20.8)

After treatment, significant improvements in symptom severity scores were seen in both groups (Table 2). The effect size (ES) of the symptom severity scores was 0.63 for both groups. However, significant improvements in functional status scores (ES 0.38) and pain scales (ES 0.74) were only seen in the US therapy group. An effect size of 0.3 to 0.8 is considered a "moderate" effect [24]. After adjusting for age, gender and baseline data, the ANCOVA analysis revealed significant differences in the functional status scores between the two study groups.

**Table 2:** **Comparison of the CTS Questionnaire and the pain scale in CTS patients**

	Treatment group		P value^b^
	Paraffin therapy	US therapy	
	(n = 23)	(n = 24)	
**Functional status score**			0.04
BT	1.7±0.6	1.8±0.8	
AT	1.8±0.9	1.6±0.7	
Difference (AT-BT)	0.1±0.9	-0.3±0.4	
Effect size	0.17	0.38	
P value^a^	0.88	0.0017	
**Symptom severity score**			0.51
BT	2.5±0.8	2.6±0.8	
AT	1.9±0.7	2.1±0.8	
Difference (AT-BT)	-0.5±0.7	-0.5±0.7	
Effect size	0.63	0.63	
P value^a^	<0.0001	0.0046	
**Pain scale**			0.81
BT	56.3±20.9	68.3±19.3	
AT	50.7±22.7	54.2±22.6	
Difference (AT-BT)	-5.7±24.1	-14.2±27.3	
Effect size	0.27	0.74	
P value^a^	0.29	0.01	

Abbreviations: CTS carpal tunnel syndrome; US ultrasound; BT before treatment; AT after treatment.

^a^Paired t test.

^b^ANCOVA comparison of differences in changes after treatment between groups after adjusting for age, gender and baseline values.

### Physical findings and NCSs

A significant improvement in the monofilament sensory test was observed in the paraffin group, and a significant improvement in the palmar pinch power test was observed in the US therapy group as well (Table 3). However, NCSs did not detect significant changes in the distal motor or sensory latencies of the median nerve in either group. Despite adjusting for baseline data, age and sex, there were no significant differences between the two study groups in any of the outcomes of the physical examinations or NCSs (Table 3).

**Table 3:** **Comparison of the results of physical examinations and NCSs in CTS patients**

	Treatment group		P value^b^
	Paraffin therapy	US therapy	
	(n = 43)	(n = 37)	
**Monofilament test**			0.95
BT	29.5±3.7	30.1±4.1	
AT	30.7±3.0	30.9±2.7	
Difference (AT-BT)	1.2±3.5	1.2±3.3	
P value^a^	0.03	0.05	
**Palmar pinch power (kg)**			0.34
BT	3.2±1.8	3.2±1.2	
AT	3.6±1.5	3.6±1.1	
Difference (AT-BT)	0.4±1.8	0.5±1.4	
P value^a^	0.44	0.01	
**Distal motor latency of the median nerve (ms)**			0.06
BT	5.10±1.27	5.11±1.34	
AT	4.98±1.51	5.08±1.30	
Difference (AT-BT)	-0.3±0.6	-0.03±0.6	
P value^a^	0.77	0.91	
**Distal sensory latency of the median nerve (ms)**			
BT	3.7±0.9	3.6±0.8	0.83
AT	3.4±0.8	3.6±1.4	
Difference (AT-BT)	-0.2±0.9	0.03±1.1	
P value^a^	0.11	0.91	

Abbreviations: CTS carpal tunnel syndrome; NCS nerve conduction study; US ultrasound; BT before treatment; AT after treatment.

^a^Paired t test (generalized estimating equation).

^b^Comparison of differences in changes after treatment between groups after adjusting for age, gender and baseline values (generalized estimating equation).

## Discussion

In this study, we found that US therapy tends to be more effective than paraffin therapy in treating CTS patients. Patients who underwent US therapy and a wrist orthosis not only experienced improvements in their functional status scores compared to those receiving paraffin therapy and a wrist orthosis but also showed statistically significant improvements in their symptom severity scores and palmar pinch power. In contrast, patients who underwent paraffin therapy and a wrist orthosis only experienced significant improvements in their symptom severity scores.

Different modes, frequencies and intensities have been used in US therapy for CTS patients [1, 3, 5, 9, 25, 26]. Generally, in US therapy, continuous mode is chosen when the thermal effect is desired, while pulsed mode is applied when the nonthermal effect is preferred [13]. Although the study conducted by Dincer et al. revealed symptom improvements after continuous mode US therapy in CTS patients [9], Oztas et al. reported a prolonged distal motor latency and a decrease in motor nerve conduction velocity after treatment with continuous mode US therapy [26]. These findings implied that though continuous mode US therapy was able to improve the symptoms in CTS patients, selective heating of the median nerve might lead to temporal conduction block [26]. On the contrary, pulsed mode US therapy effectively enhanced peripheral nerve regeneration in an animal study, possibly through the mechanisms of local blood vessel dilatation, nerve sprouting stimulation, Schwann cell activation and chemotactic stimulator release [27].

This study utilized pulsed mode US therapy on CTS patients and observed improvements in subjective symptoms and palmar pinch power, similar to previous studies [1, 3]. However, we did not note significant improvements in distal motor and sensory latencies of the median nerve after eight weeks of treatment. These findings corroborate studies conducted by Yildiz et al. and Baysal et al., who were also unable to find significant improvement in distal latencies of the median nerve in CTS patients after applying pulsed mode US therapy and followed up for eight weeks [3, 25]. This negative result might be because A fibers in the peripheral nerve system are measured mostly in clinical NCSs, but C fibers, which transmit somatic pain signals, are more sensitive to US effects than A fibers [6, 28, 29]. This difference might explain the fact that, despite significant symptom improvements in our study, NCSs did not detect significant improvements in distal motor and sensory latencies of the median nerve. Moreover, delayed recovery of nervous tissue could contribute to the lack of improvement seen in NCSs. As shown in Harris et al.’s study, CTS patients who underwent surgical decompression experienced delayed electrophysiological recovery of up to six months [30]. Inadequate follow-up time may underestimate the electrophysiological improvement; thus, further study with a longer follow-up time is recommended.

Paraffin therapy is a superficial heat physical agent that uses conduction to transfer heat. Its therapeutic effects include increasing blood flow, producing analgesic effects, decreasing chronic inflammation, improving connective tissue elasticity and stimulating general muscle relaxation [6, 31]. In this study, patients receiving a combination treatment of paraffin therapy and a wrist orthosis exhibited improvements in symptom severity scores and in the monofilament sensory test, consistent with a previous study [4]. These findings could be regarded as a validation of the baseline measurements of this trial. In the US therapy group, a significant improvement in pinch power was noted, in addition to symptom improvements, which further improved patients' functional status. This result might be partially contributed by the nonthermal effect of pulsed US therapy.

Though it requires more manpower to implement US therapy than paraffin therapy, combination treatment with US therapy and a wrist orthosis is recommended because of its superior effect on functional status and possibly on nerve regeneration. Further study to compare the effectiveness of pulsed vs. continuous US therapy in CTS patients is suggested.

This study has several limitations. First, because this study was performed in the Department of Physical Medicine and Rehabilitation, the patients usually suffered from mild to moderate symptoms. Therefore, we should remain cautious in our attempts to generalize our findings to patients with more severe symptoms. Second, this study used a combination treatment of a wrist orthosis with either US or paraffin therapy because it would have been unethical to withhold wrist orthoses when they have been reported to be effective [9]. Therefore, the treatment effects might partially originate from the wrist orthoses. Third, because approximately 20% of the participants did not complete this study, we could not perform the intention-to-treat analyses. To examine the potential bias caused by loss of follow-up, we compared the demographic and baseline symptoms severity scales, functional status scores and pain scales between the patients who completed the study and those who did not. The results revealed no significant differences between the follow-up and non-follow-up groups. Thus, we believe the potential bias may be minimal because all patients were instructed in the same manner and were randomized into two different groups. Fourth, because this study compared the two studied groups regarding their functions, symptoms, pain and results on four clinical tests, we were concerned about multiplicity issues. Of the 7 outcomes evaluated, only the primary outcome (functional status score) exhibited a significant difference between the two studied groups. Thus, further randomized, controlled trials with long-term follow-ups could be needed to validate these results.

## Conclusions

To improve the functional status of CTS patients, a combination of ultrasound therapy and a wrist orthosis may be more effective than a combination of paraffin therapy and a wrist orthosis. Since this is an exploratory trial, further confirmatory testing is suggested to justify the efficacy of these two treatments.

## Authors’ original submitted files for images

Below are the links to the authors’ original submitted files for images. Authors’ original file for figure 1

## Abbreviations

CTS: Carpal tunnel syndrome
US therapy: Ultrasound therapy
NCSs: Nerve conduction studies
VAS: Visual analog scale
PRO: Patient-reported outcomes
ANCOVA: Analysis of covariance
GEE: Generalized estimate equation.
