# Supplementary material for: Comparative effectiveness of ultrasound and paraffin therapy in patients with carpal tunnel syndrome: a randomized trial
Source: BMC Musculoskelet Disord. 2014 Nov 26;15:399. doi: 10.1186/1471-2474-15-399 (PMC4256823; doi:10.1186/1471-2474-15-399)
Supplement: Supplementary file 1 — Authors’ original file for figure 1 [file 12891_2014_2335_MOESM1_ESM.pdf]

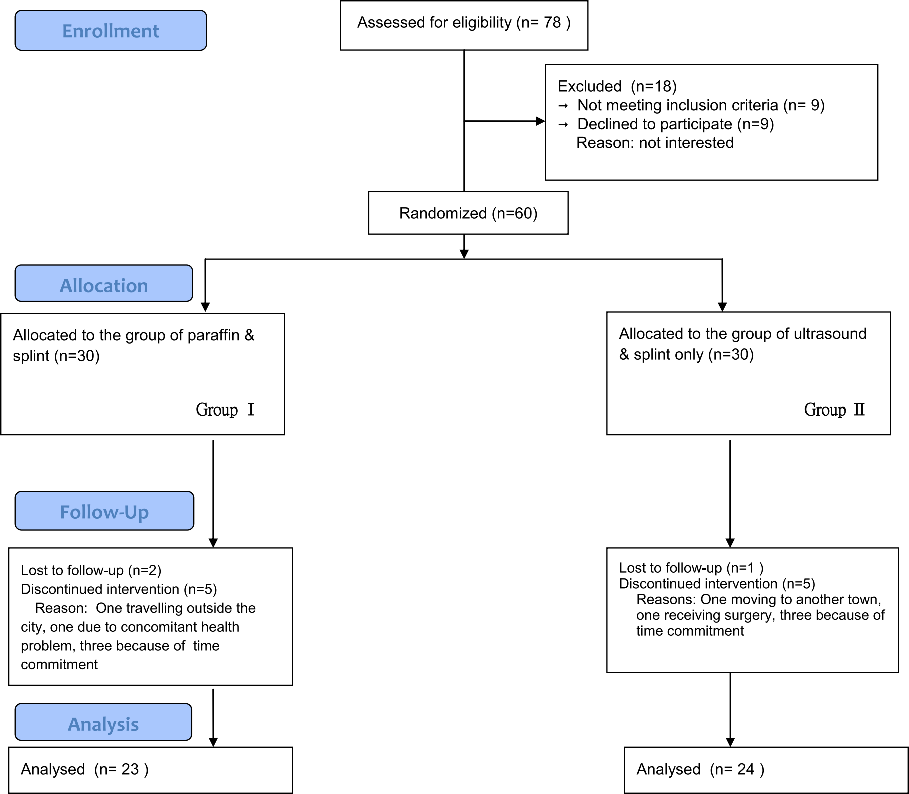
Enrollment
Assessed for eligibility (n= 78)
Excluded (n=18)
→ Not meeting inclusion criteria (n= 9)
→ Declined to participate (n=9)
Reason: not interested
Randomized (n=60)
Allocation
Allocated to the group of paraffin & splint (n=30)
Group I
Allocated to the group of ultrasound & splint only (n=30)
Group II
Follow-Up
Lost to follow-up (n=2)
Discontinued intervention (n=5)
Reason: One travelling outside the city, one due to concomitant health problem, three because of time commitment
Lost to follow-up (n=1)
Discontinued intervention (n=5)
Reasons: One moving to another town, one receiving surgery, three because of time commitment
Analysis
Analysed (n= 23)
Analysed (n= 24)
